# Supplementary material for: The unusual structural properties and potential biological relevance of switchback DNA
Source: Nat Commun. 2024 Aug 6;15:6636. doi: 10.1038/s41467-024-50348-3 (PMC11303717; doi:10.1038/s41467-024-50348-3)
Supplement: Supplementary file 1 — Supplementary information [file 41467_2024_50348_MOESM1_ESM.pdf]

Supplementary Information

The unusual structural properties and potential biological relevance of switchback DNA

Bharath Raj Madhanagopal,[1] Hannah Talbot,[1] Arlin Rodriguez,[1] Jiss Maria Louis,[1] Hana Zeghal,[1] Sweta Vangaveti,[1] Kaalak Reddy,[1,2] and Arun Richard Chandrasekaran[1,3]*

[1]The RNA Institute, University at Albany, State University of New York, Albany, NY, USA.

[2]Department of Biological Sciences, University at Albany, State University of New York, Albany, NY, USA.

[3]Department of Nanoscale Science and Engineering, University at Albany, State University of New York, Albany, NY, USA.

*Correspondence: arun@albany.edu

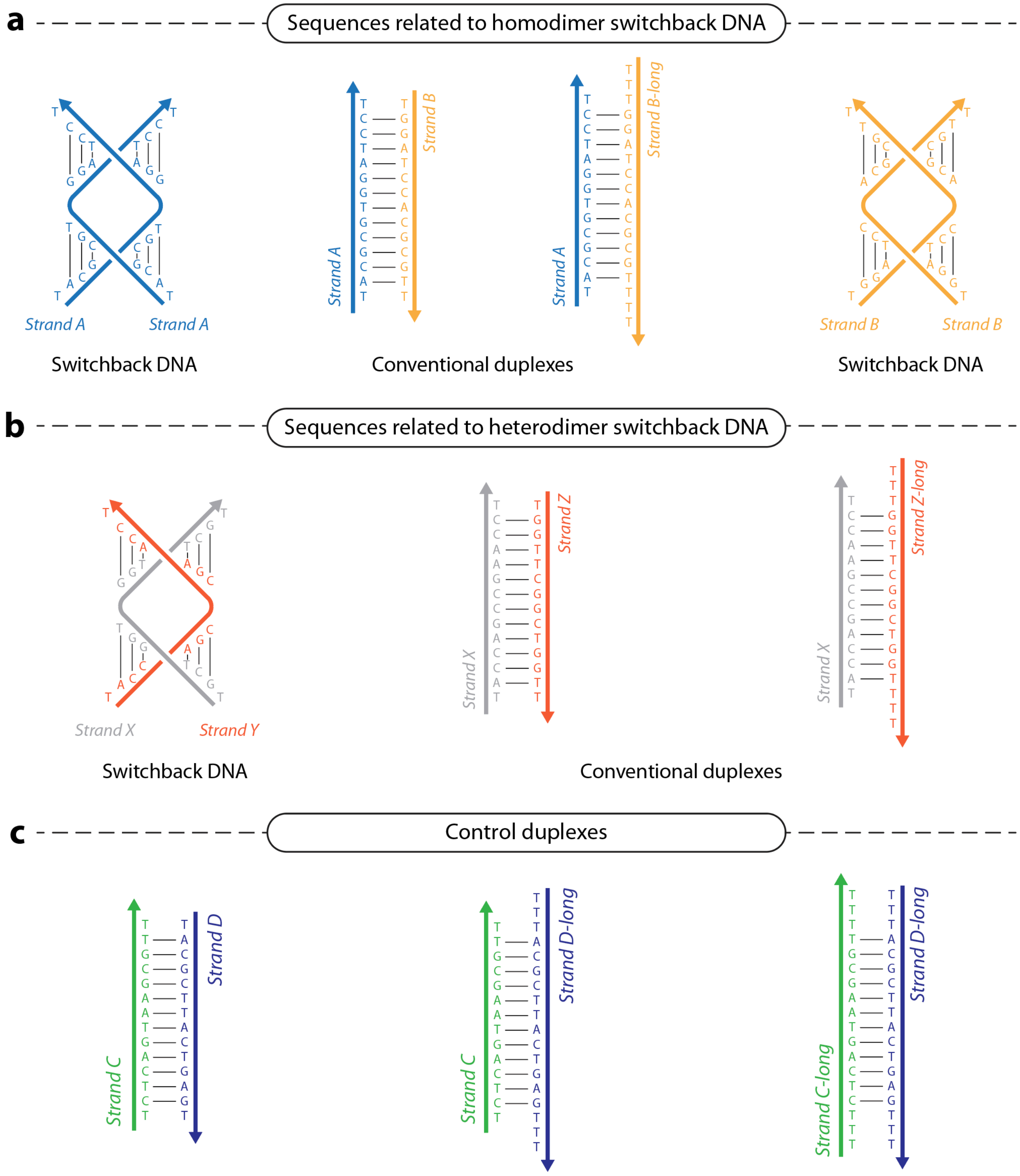

Supplementary Fig. 1. Sequences of DNA complexes used in this study. (**a**) Homodimeric switchback DNA and its corresponding conventional duplexes. (**b**) Heterodimeric switchback DNA and its corresponding conventional duplexes. (**c**) Control structures.

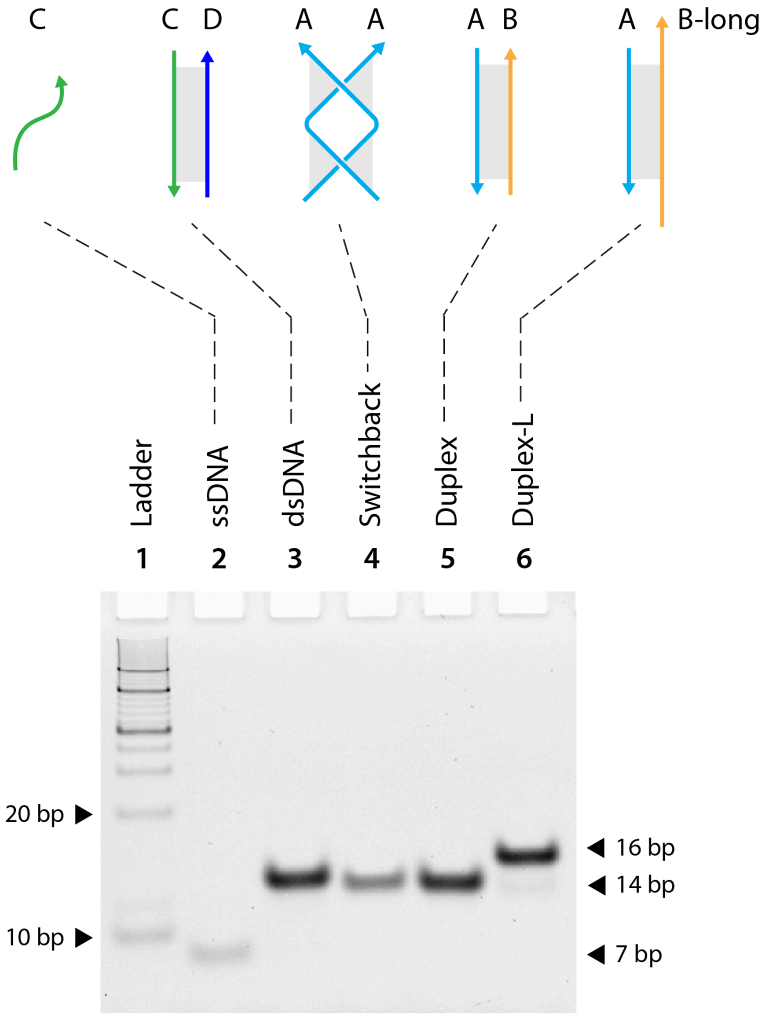

Supplementary Fig. 2. Non-denaturing gel showing the assembly of homodimer switchback and its corresponding duplex. Full image of the gel shown in Fig. 2a. The data shown is representative of experiments performed multiple times (n > 3) with similar reproducible results.

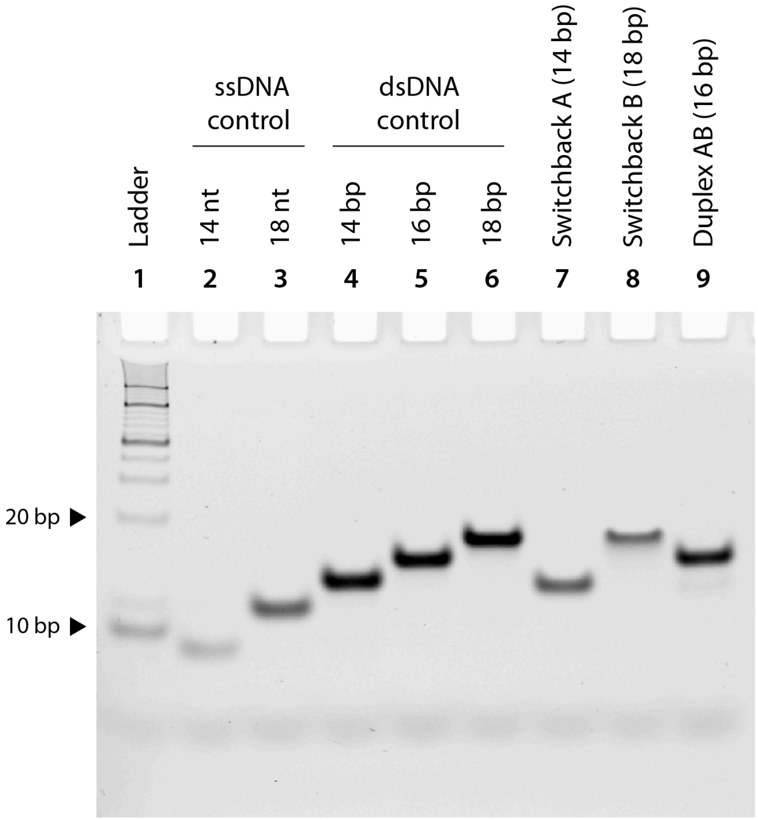

Supplementary Fig. 3. Non-denaturing gel showing the assembly of homodimer switchback, corresponding duplex, and control structures. The data shown is representative of experiments performed multiple times (n > 3) with similar reproducible results.

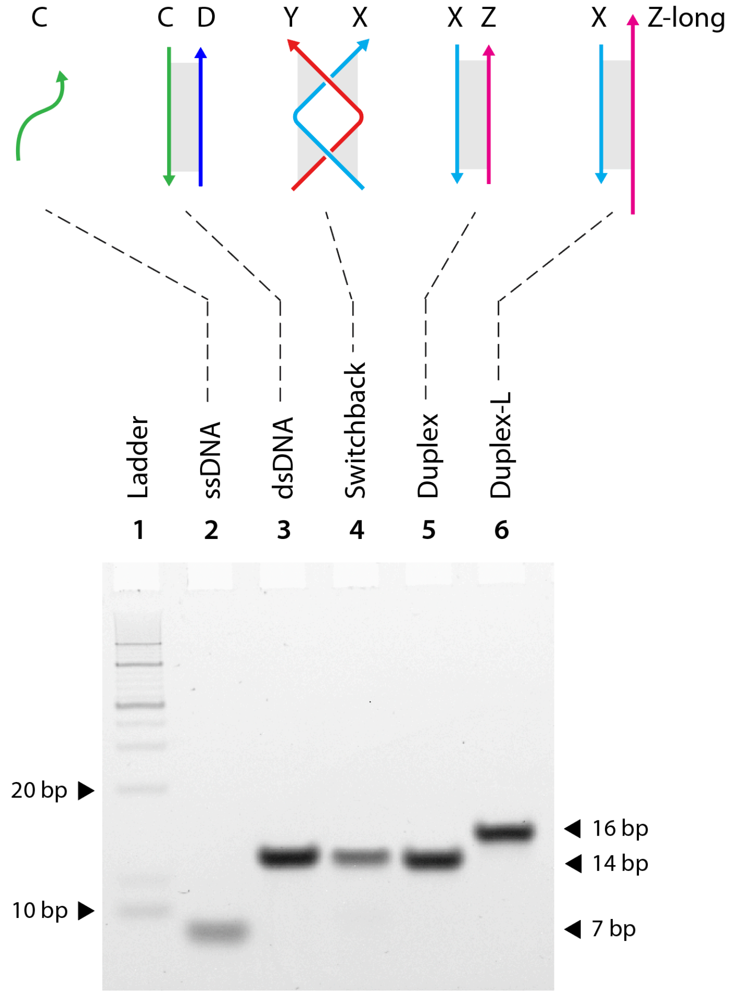

Supplementary Fig. 4. Non-denaturing gel showing the assembly of heterodimer switchback and its corresponding duplex. Full image of the gel shown in Fig. 2b. The data shown is representative of experiments performed multiple times (n > 3) with similar reproducible results.

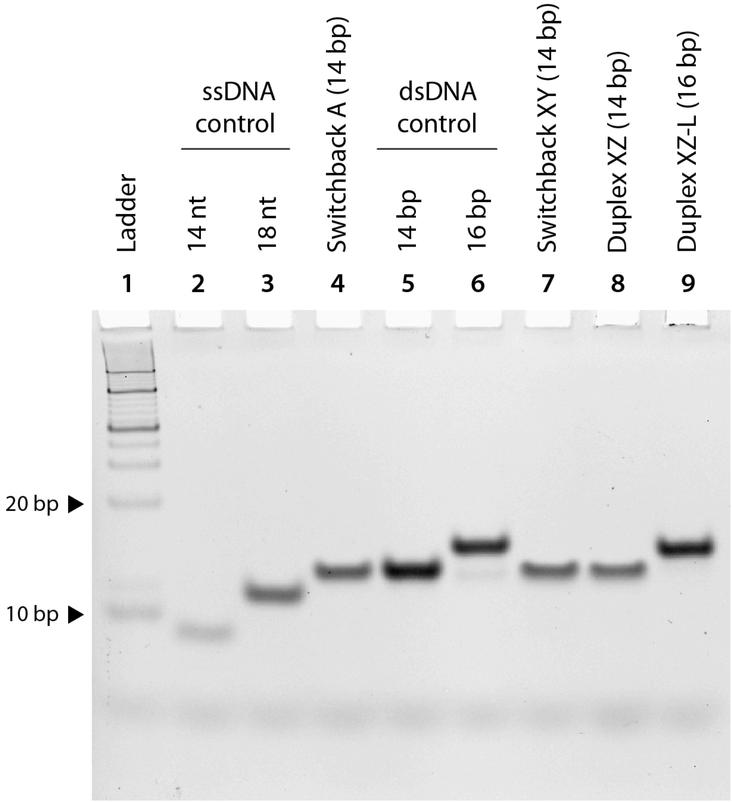

Supplementary Fig. 5. Non-denaturing gel showing the assembly of heterodimer switchback, corresponding duplex, and control structures. The data shown are representative of experiments performed multiple times (n > 3) with similar reproducible results.

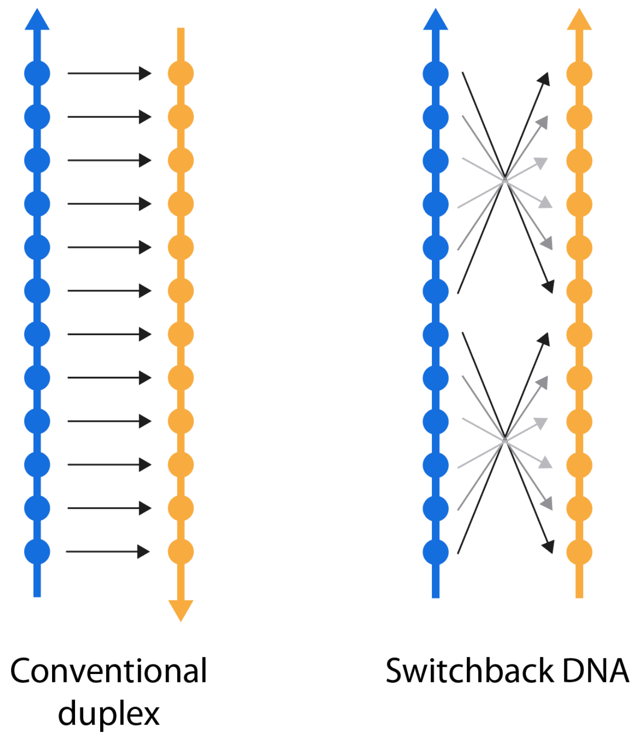

Supplementary Fig. 6. Base pairing rules for conventional duplexes and switchback DNA. Shown here is a structure with two half-turn domains. The black and grey arrows map the pairs of complementary bases.

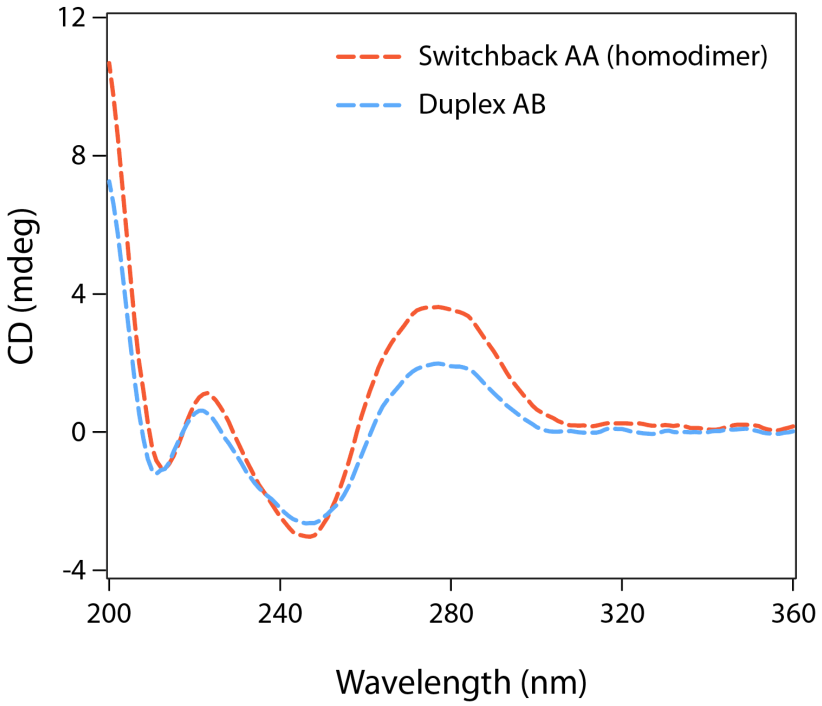

Supplementary Fig. 7. CD spectra of homodimeric switchback DNA and its corresponding conventional duplex. The data shown are representative of experiments performed twice (n = 2) with similar reproducible results.

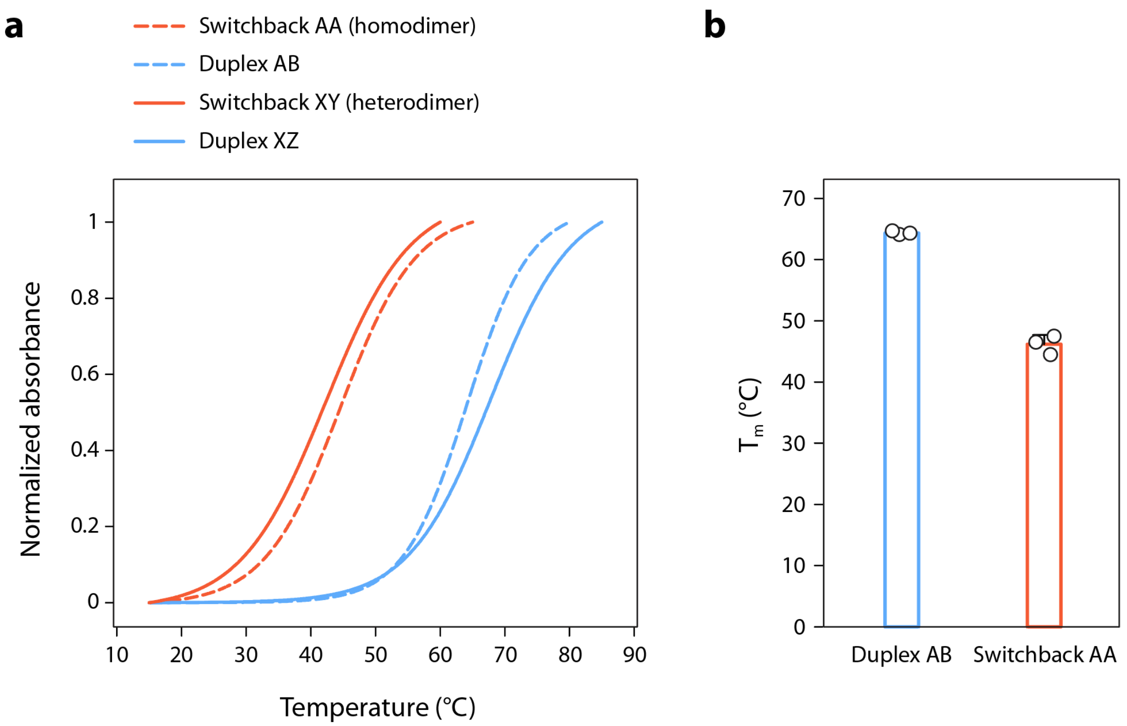

Supplementary Fig. 8. (**a**) UV melting curves of switchback DNA and conventional duplexes. (**b**) Melting temperatures (Tm) of homodimer switchback DNA and the corresponding conventional duplex. Data represent mean and error propagated from standard deviations of experiments performed in triplicates (n = 3 independent experiments).

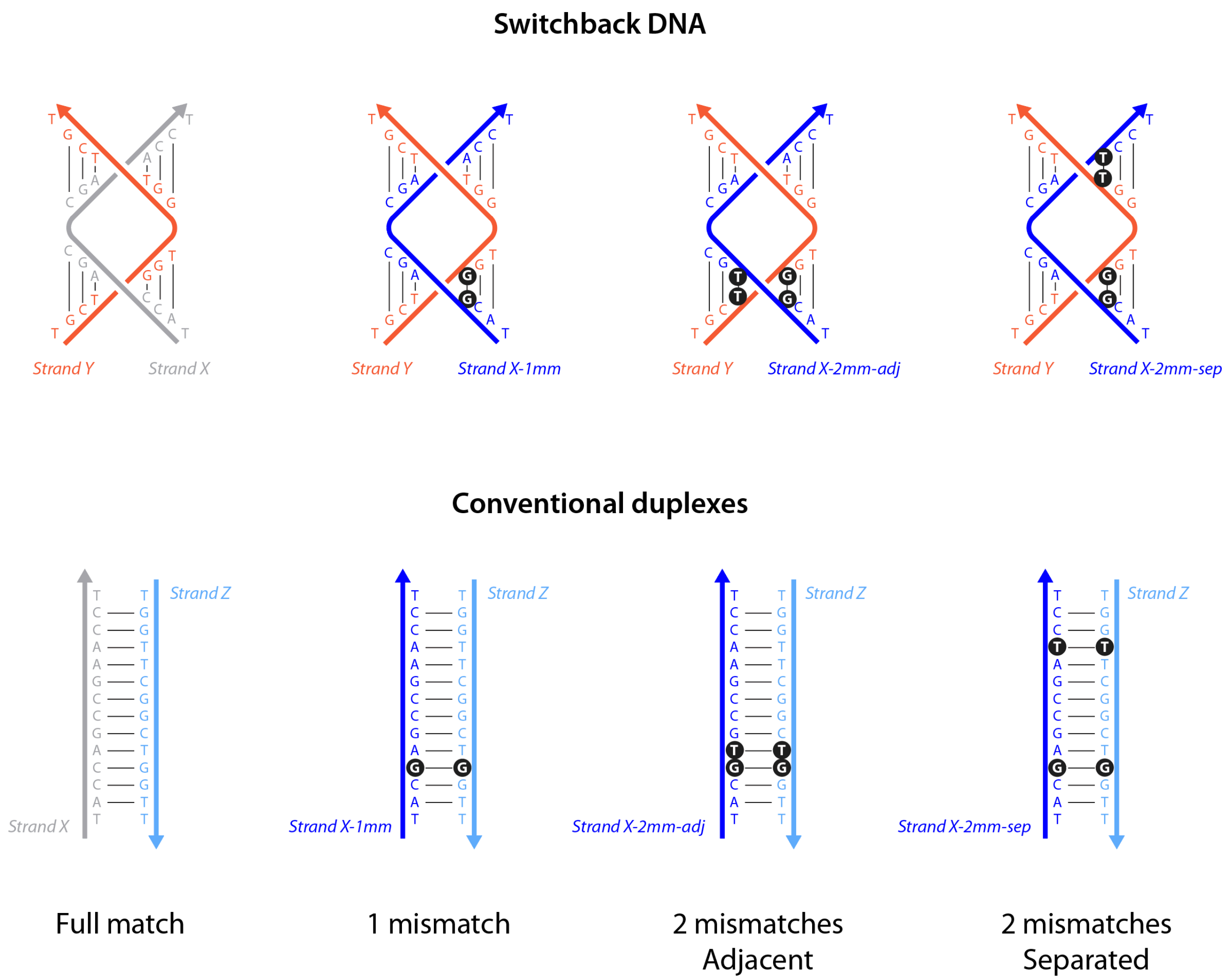

Supplementary Fig. 9. Design of switchback DNA and conventional duplexes with mismatched base pairs.

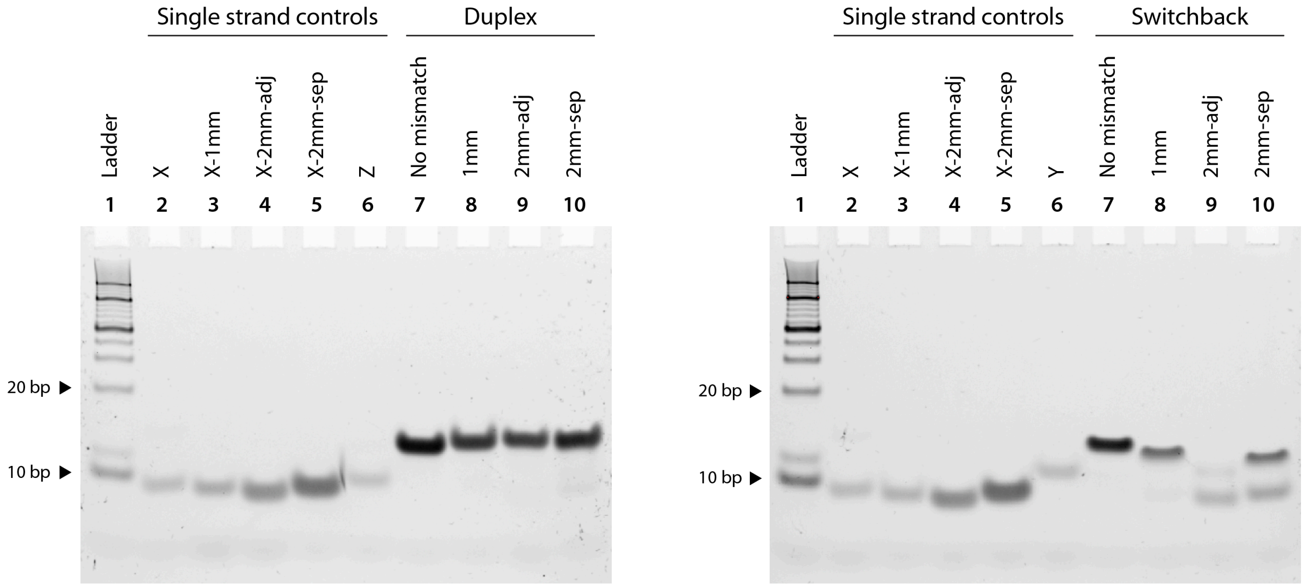

Supplementary Fig. 10. Non-denaturing polyacrylamide gel image of conventional duplex and switchback DNA with mismatched base pairs. The data shown are representative of experiments performed multiple times (n > 3) with similar reproducible results.

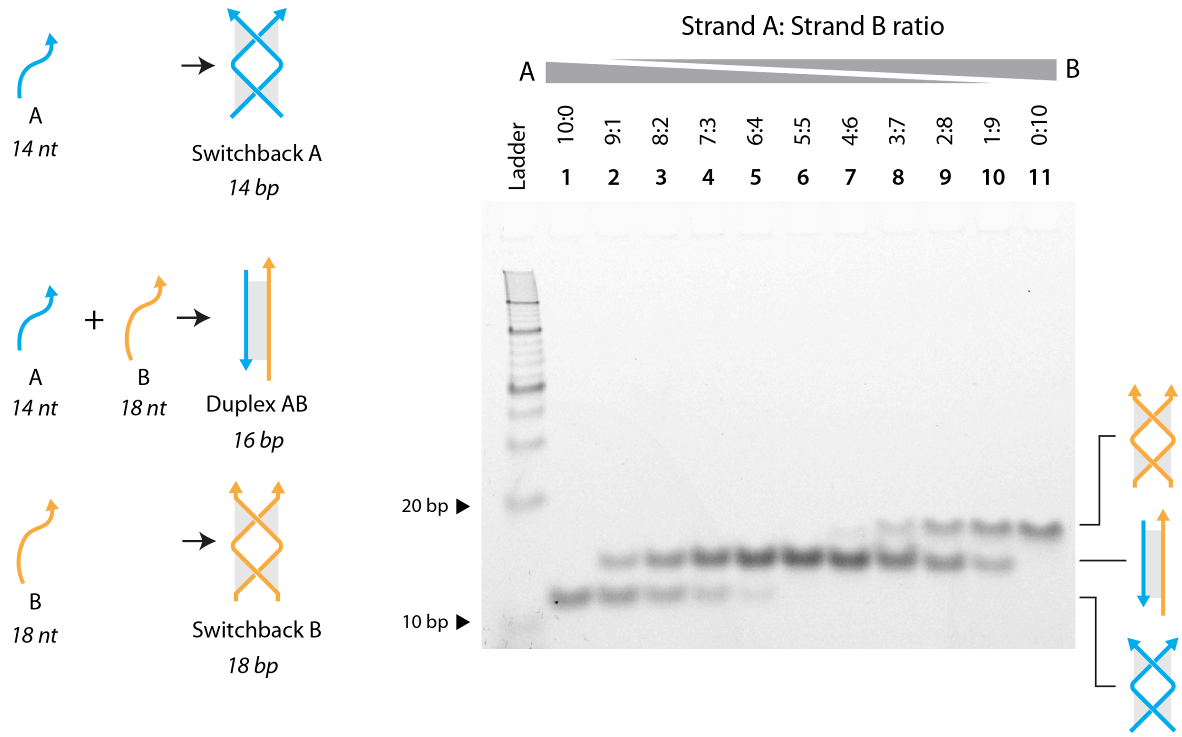

Supplementary Fig. 11. Strand competition between switchback DNA and conventional duplex in homodimer assembly. Full image of the gel shown in Fig. 3a. The data shown is representative of experiments performed in triplicates (n = 3 independent experiments).

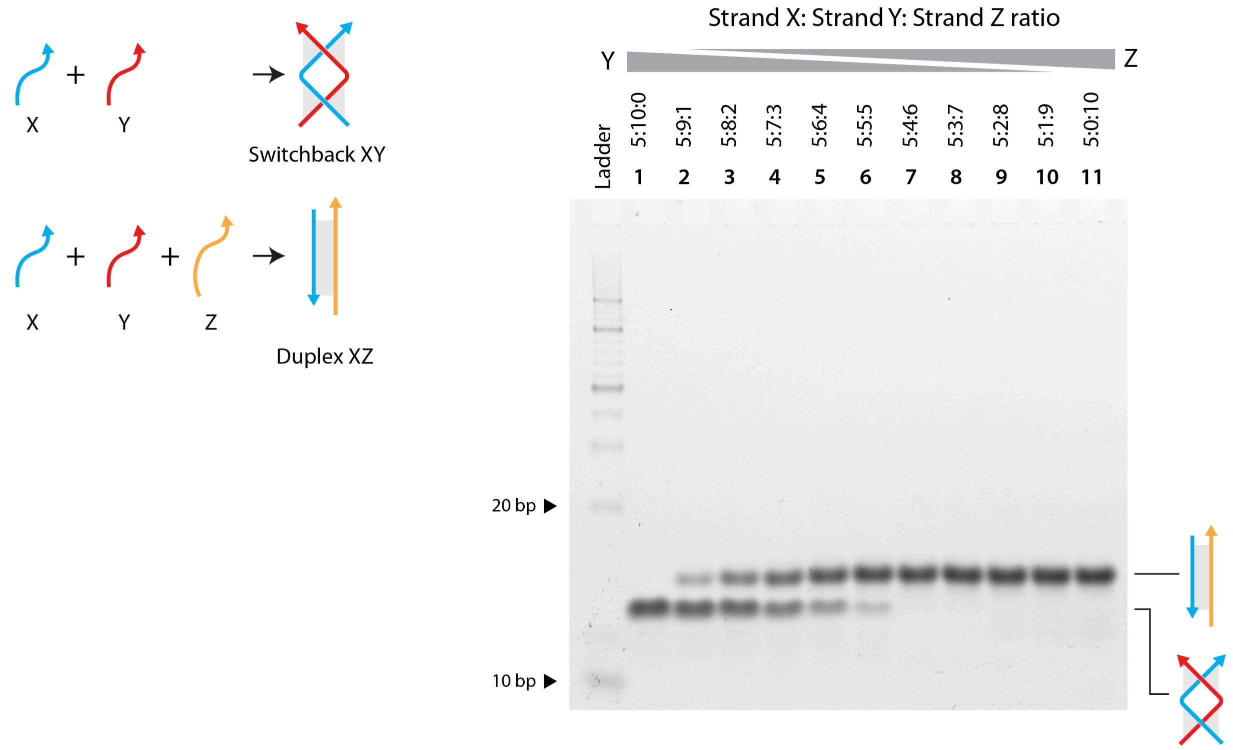

Supplementary Fig. 12. Strand competition between switchback DNA and conventional duplex in heterodimer assembly. Full image of the gel shown in Fig. 3b. The data shown is representative of experiments performed in triplicates (n = 3 independent experiments).

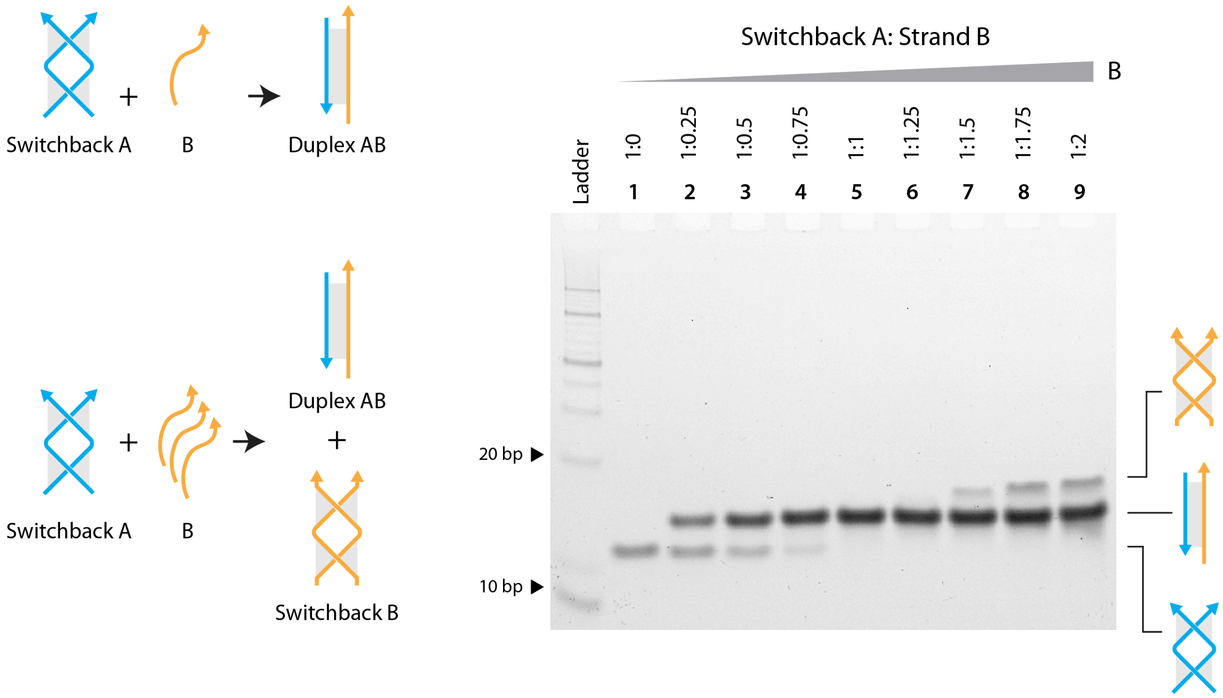

Supplementary Fig. 13. Strand displacement in homodimer switchback DNA on the addition of a duplex complement. Full image of gel shown in Fig. 3c. The data shown is representative of experiments performed in triplicates (n = 3 independent experiments).

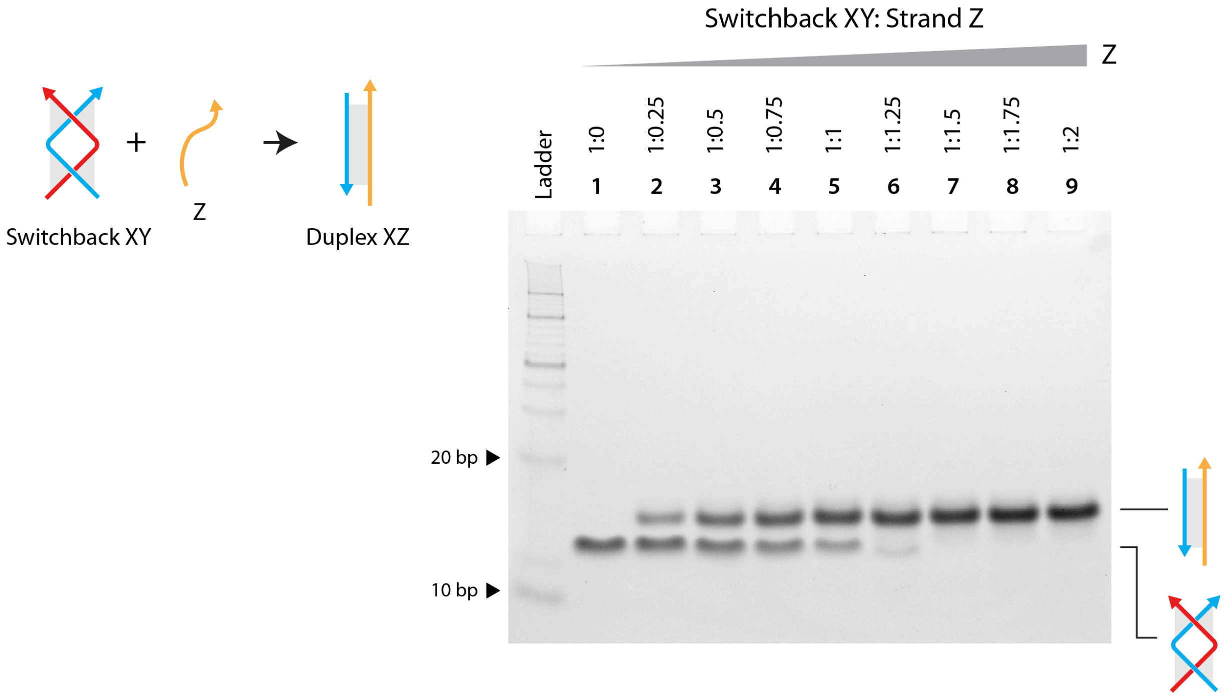

Supplementary Fig. 14. Strand displacement in heterodimer switchback DNA on the addition of a duplex complement. Full image of gel shown in Fig. 3d. The data shown is representative of experiments performed in triplicates (n = 3 independent experiments).

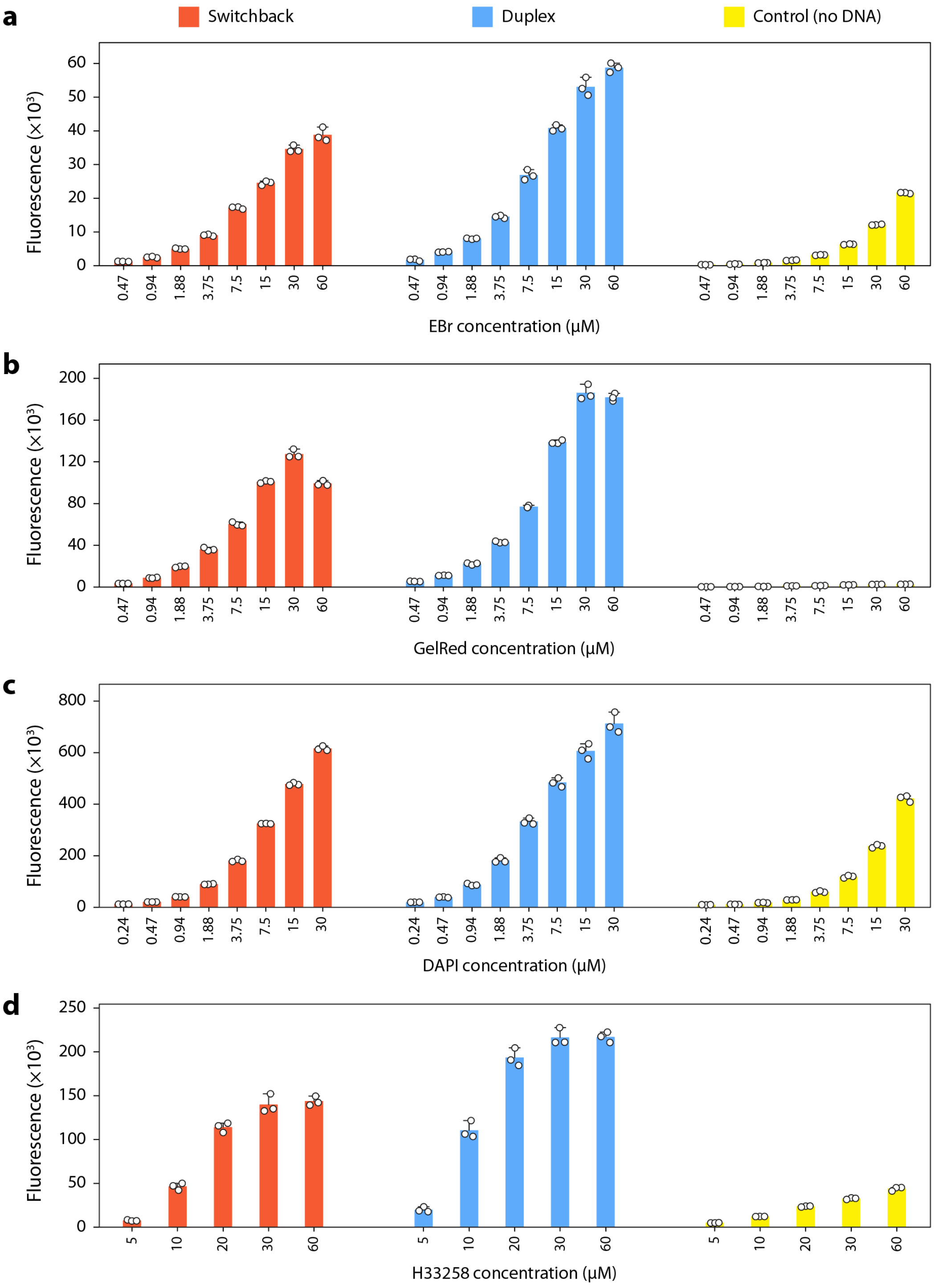

Supplementary Fig. 15. Raw fluorescence signals of (**a**) ethidium bromide (EBr), (**b**) GelRed, (**c**) 4′6-diamidino-2-phenylindole (DAPI), (**d**) Hoechst 33258 (H33258) with switchback DNA XY (red), duplex DNA XZ (blue) and without any DNA (yellow). Data represent mean and error propagated from standard deviations of experiments performed in triplicates (n = 3 independent experiments).

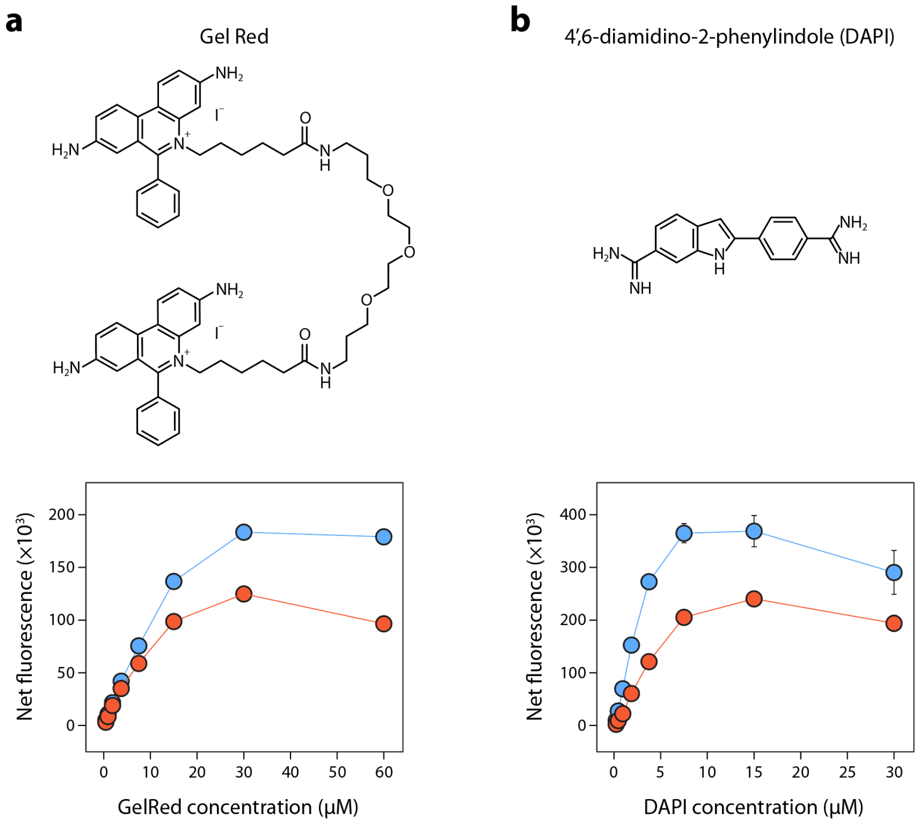

Supplementary Fig. 16. Fluorescence intensities of switchback DNA and conventional duplexes with different concentrations of (**a**) GelRed (intercalator) and (**b**) DAPI (minor groove binder). Data represent mean and error propagated from standard deviations of experiments performed in triplicates (n = 3 independent experiments).

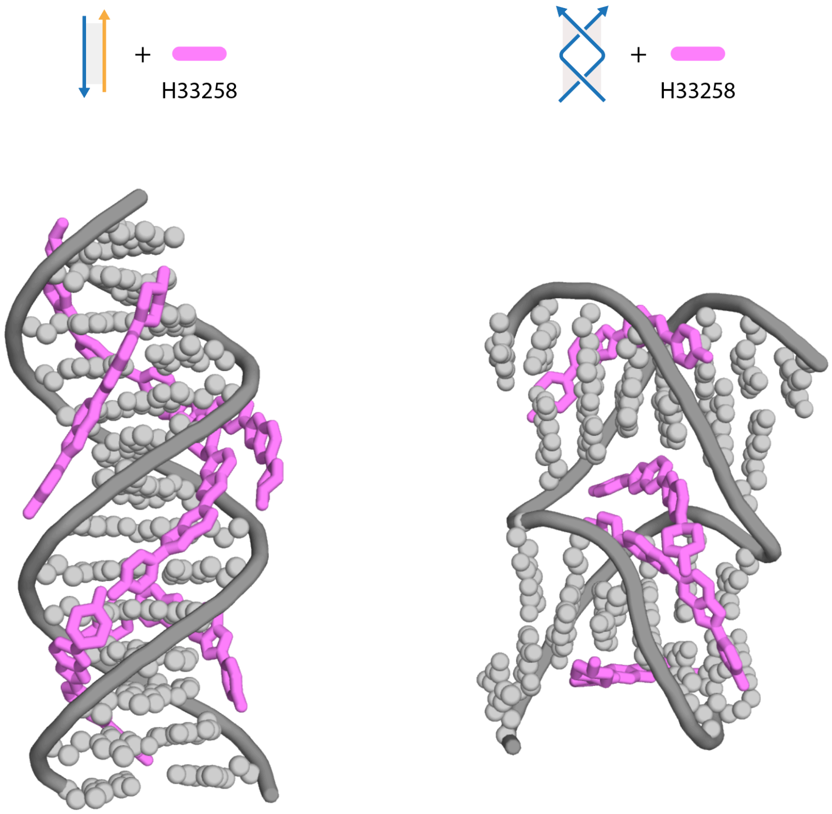

Supplementary Fig. 17. Molecular docking analysis of conventional duplex and switchback DNA with H33258 respectively. We sequentially docked H33258 on the duplex or switchback DNA until the number of predicted contacts between the ligand and DNA in a docking run was less than two.

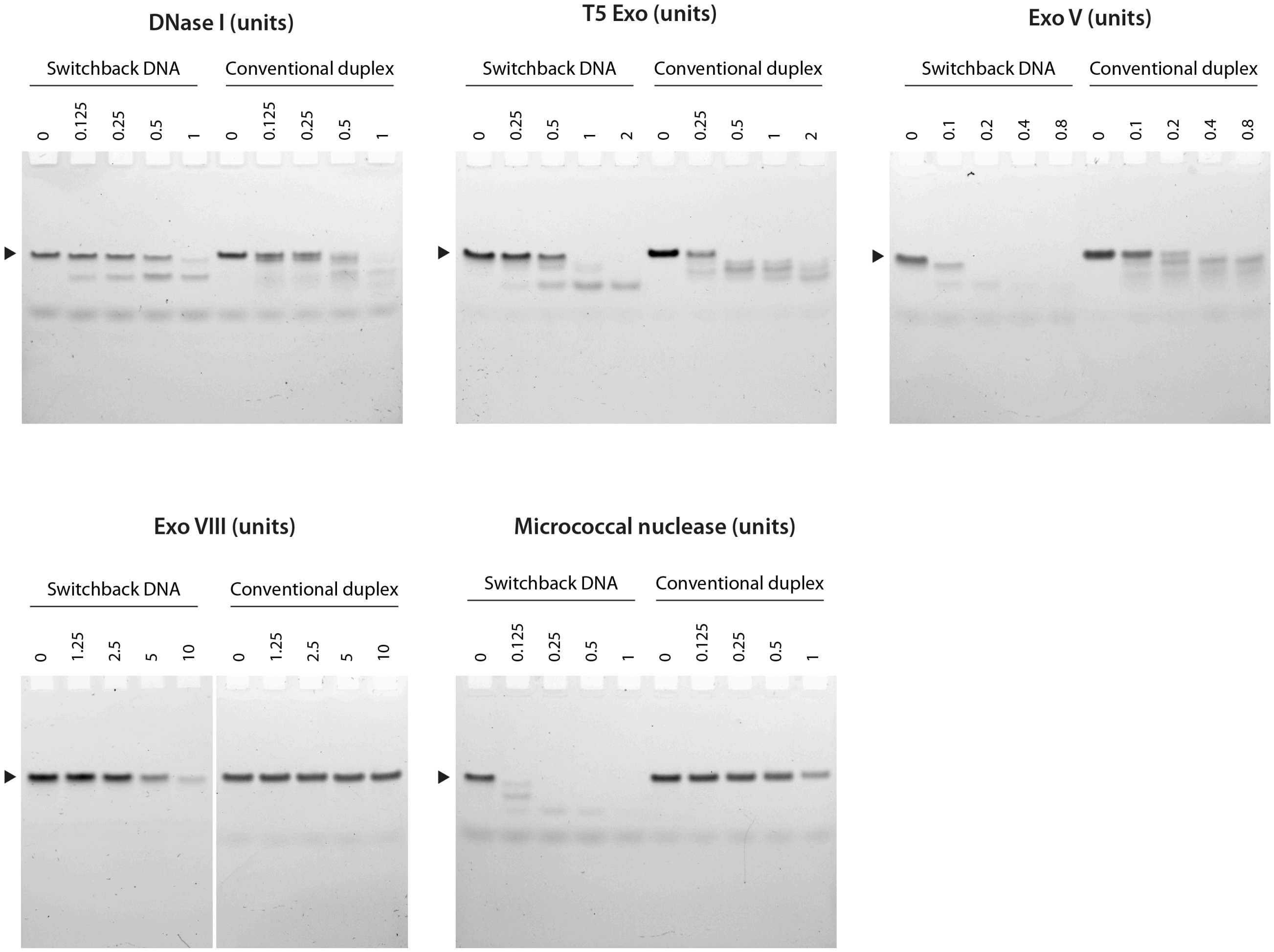

Supplementary Fig. 18. Non-denaturing gel images of switchback DNA and conventional duplex treated with different amounts of various nucleases. The data shown are representative of experiments performed in triplicates (n = 3 independent experiments).

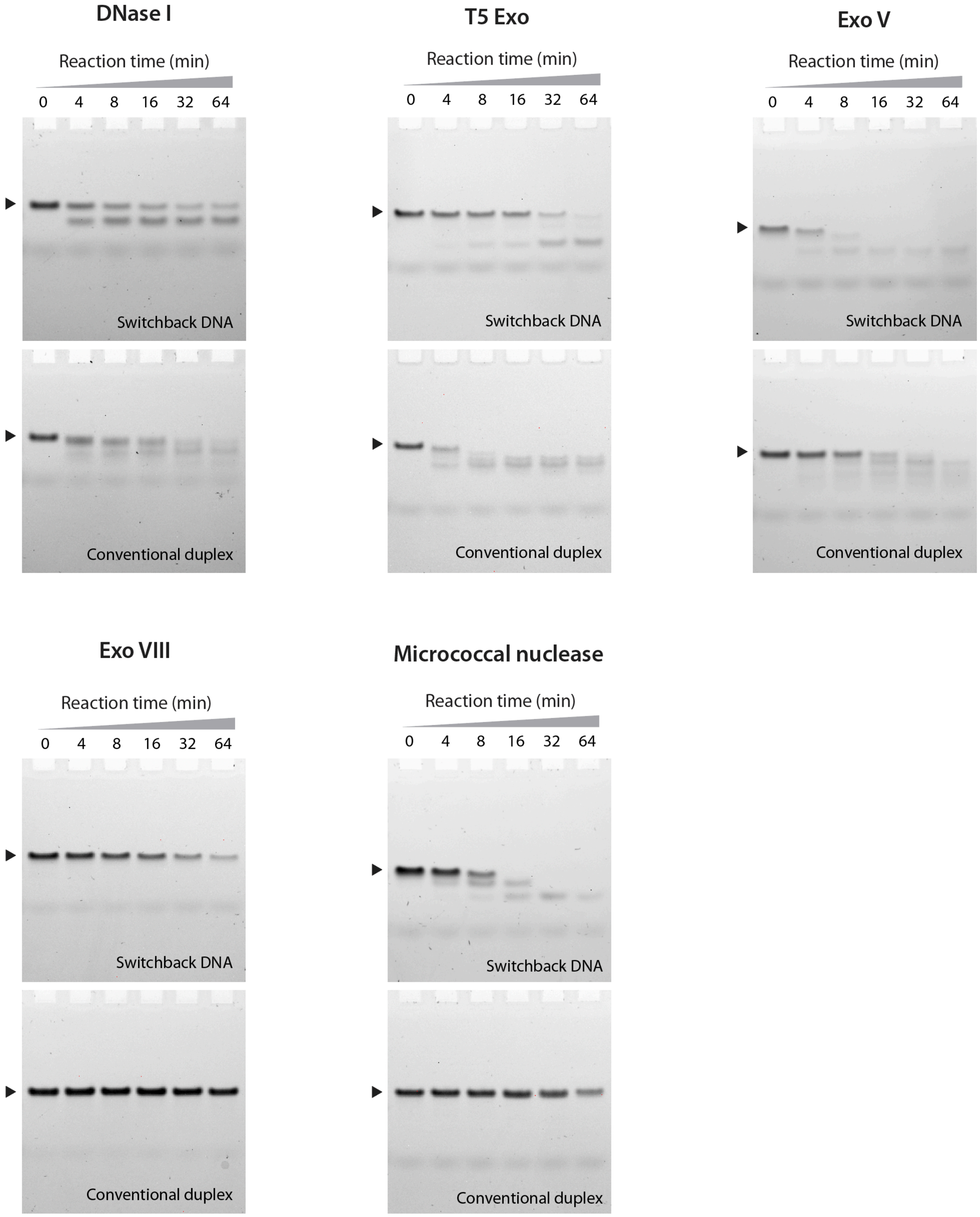

Supplementary Fig. 19. Non-denaturing gel images of switchback DNA and conventional duplex treated with various nucleases for different time periods (DNase I: 0.75 U, T5 Exo: 1 U, Exo V: 0.5 U, Exo VIII: 10 U, micrococcal nuclease: 0.25 U). The data shown are representative of experiments performed with a minimum of two replicates (n ≥ 2 independent experiments).

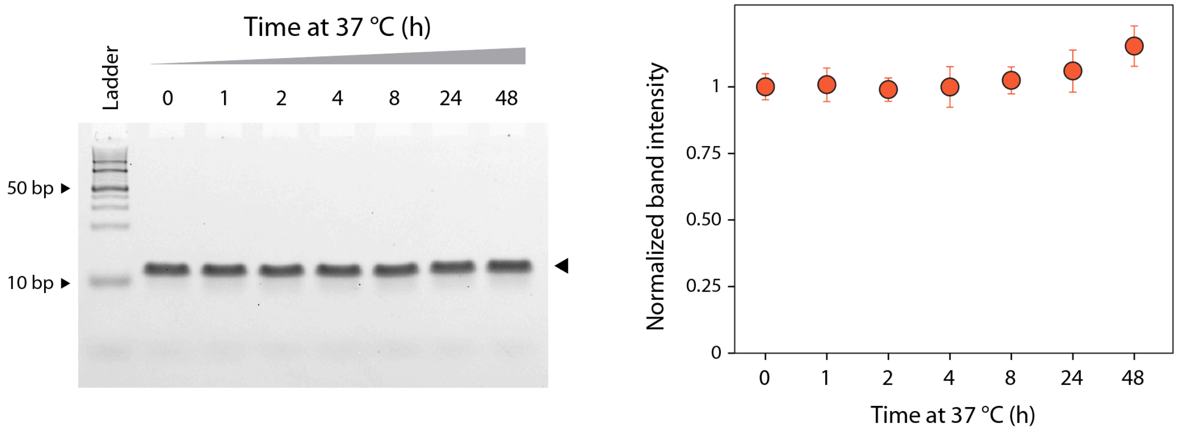

Supplementary Fig. 20. Representative non-denaturing gel image showing the stability of switchback DNA XY at 37 °C for up to 48 hours (left). Change in the intensity of the switchback DNA band with incubation time (right). Data represent mean and error propagated from standard deviations of experiments performed in triplicates (n = 3 independent experiments).

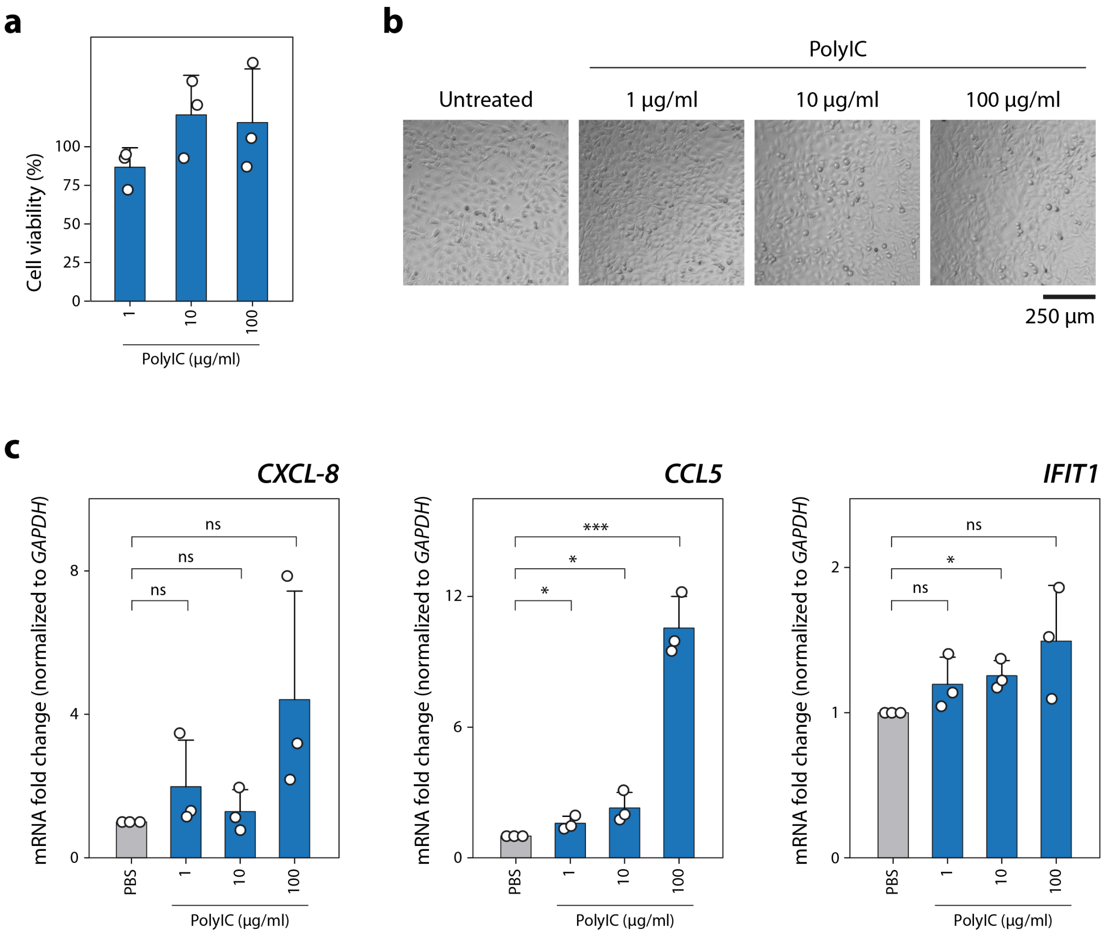

Supplementary Fig. 21. (**a**) Cell viability in HeLa cells incubated with different concentrations of polyIC. (**b**) Representative microscopy images of untreated cells and cells treated with polyIC. (**c**) RT-qPCR analysis of immune response markers, *CXCL-8* (From left-to-right: *P*= 0.262; *P*= 0.4544; *P*= 0.1226), *CCL5* (from left-to-right: *P*= 0.0344; *P*= 0.0368; *P*= 0.0003), and *IFIT1* (from left-to-right: *P*= 0.1459; *P*= 0.0135; *P*= 0.0882). Data represent mean and standard deviation from biological triplicates (n = 3 independent experiments). Unpaired two-tailed *t*-test was used to compare polyIC treatment to PBS control; ns – not significant, *$P < 0.05$, ***$P < 0.001$.

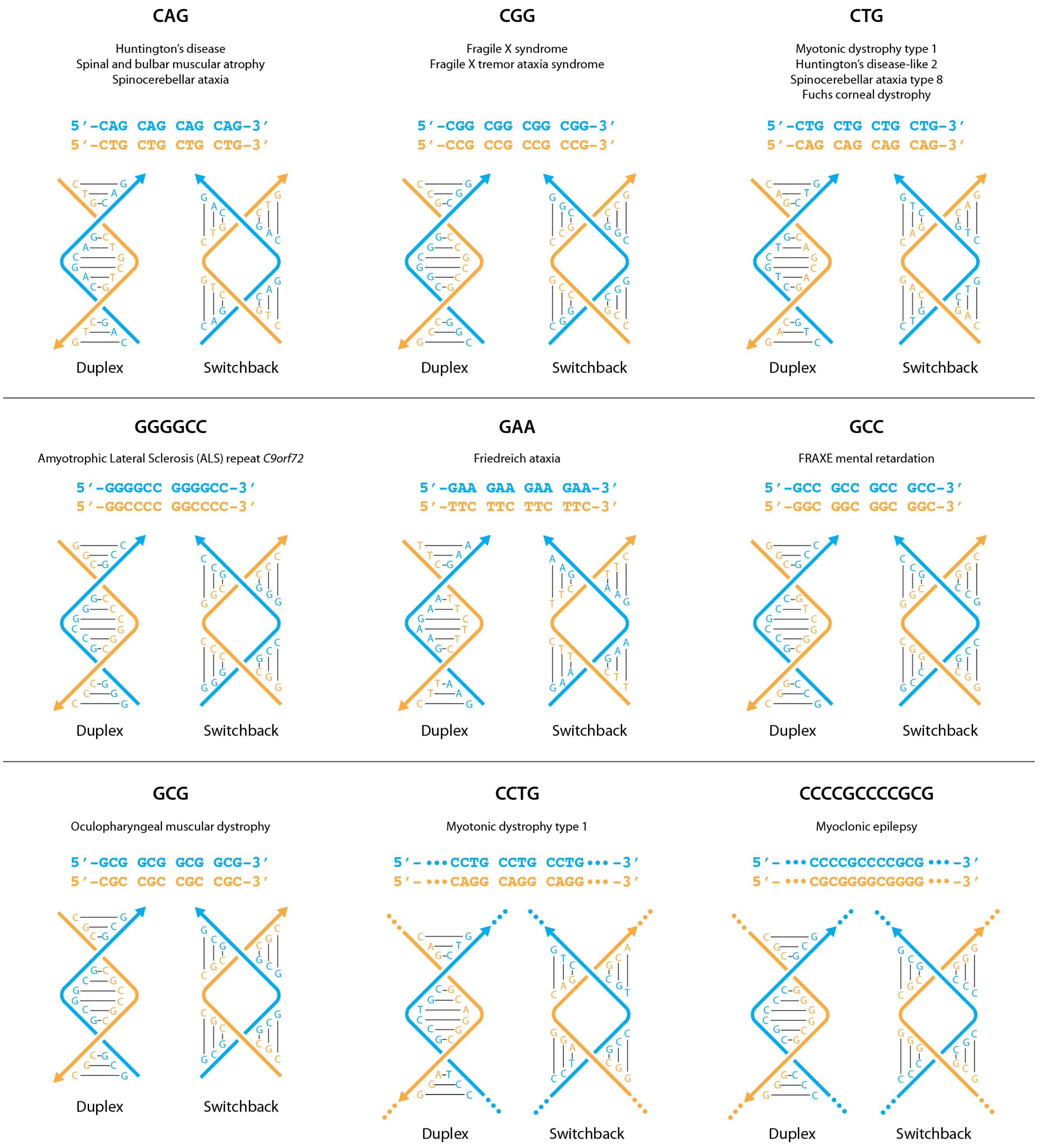

Supplementary Fig. 22. Select repeat sequences involved in various diseases with the sense and antisense strands in a typical duplex form and the predicted switchback DNA form.

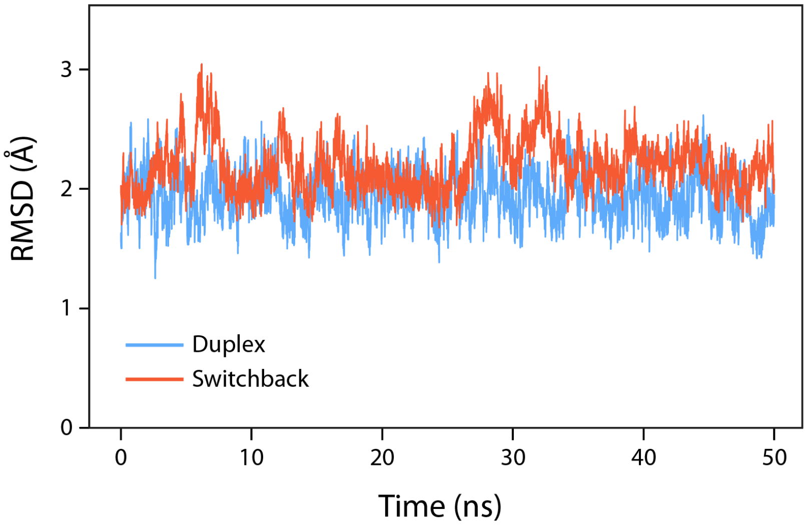

Supplementary Fig. 23. Root mean square deviation (RMSD) of the conventional duplex and switchback DNA as a function of time. The data shown are representative of experiments performed multiple times (n = 3) with similar reproducible results.

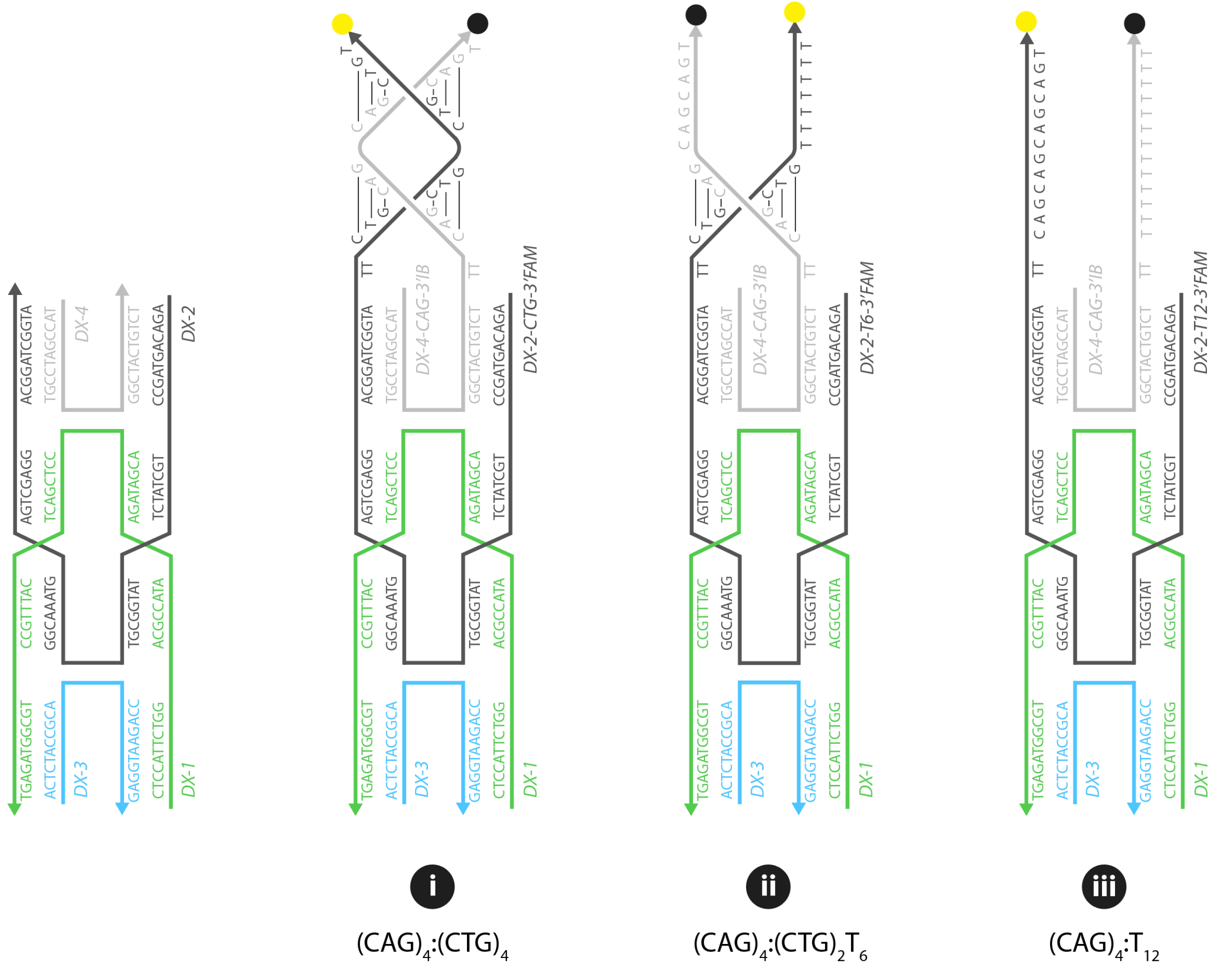

Supplementary Fig. 24. Design and sequences used in the DX scaffold.

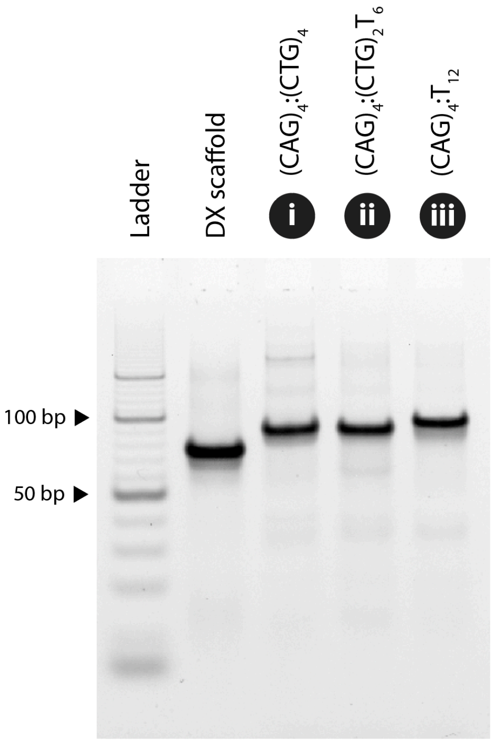

Supplementary Fig. 25. Non-denaturing PAGE analysis of DX scaffold containing different interacting regions. Full image of gel shown in Fig. 7g. The data shown is representative of experiments performed twice (n = 2) with similar reproducible results.

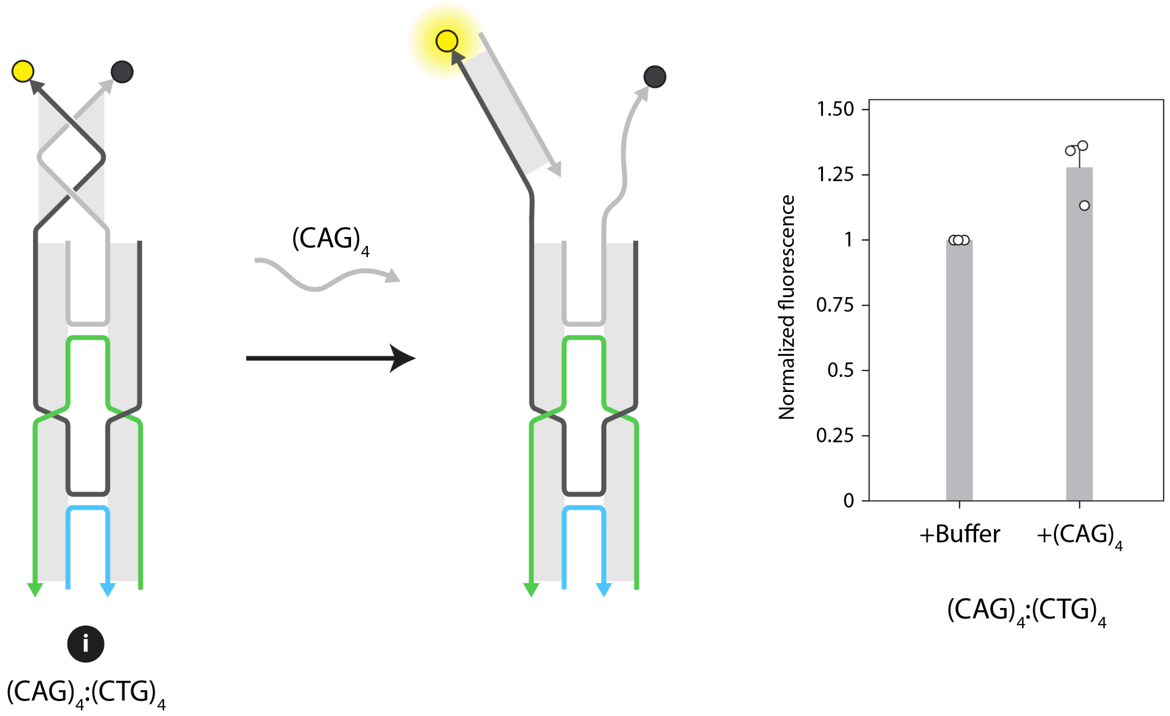

Supplementary Fig. 26. Fluorescence analysis of the interaction between two regions on the DX scaffold on the addition of a duplex complement. Data represent mean and error propagated from standard deviations of experiments performed in triplicates (n = 3 independent experiments).

Supplementary Table 1. Sequences used in this study. Strand combinations for different structures are shown in Supplementary Fig. 1, 9 and 24.

Name	Sequence (5′-3′)	Length
Homodimer switchback and corresponding duplex		
A	TACGCGTGGATCCT	14
B	TGGATCCACGCGTT	14
B-long	TTTGGATCCACGCGTTTT	18
Heterodimer switchback and corresponding duplex		
X	TACCAGCCGAACCT	14
Y	TGCTGGTGGTTCGT	14
Z	TGGTTCGGCTGGTT	14
Z-long	TTTGGTTCGGCTGGTTTT	18
X-5′FAM	/56-FAM/TACCAGCCGAACCT	14
Y-5′IB	/5IABkFQ/TGCTGGTGGTTCGT	14
Z-5′IB	/5IABkFQ/TGGTTCGGCTGGTT	14
Heterodimer switchback and corresponding duplex with mismatches		
X-1m	TACGAGCCGAACCT	14
X-2mm-adj	TACGTGCCGAACCT	14
X-2mm-sep	TACGAGCCGATCCT	14
Control single strands and duplexes		
C	TCTCAGTAAGCGTT	14
D	TACGCTTACTGAGT	14
C-long	TTTCTCAGTAAGCGTTTT	18
D-long comp	TTTACGCTTACTGAGTTT	18
Double crossover scaffold		
DX-1	CTCCATTCTGGACGCCATAAGATAGCACCTCGACTCATTTGCCTGCGGTAGAGT	54
DX-2	AGACAGTAGCCTGCTATCTTATGGCGTGGCAAATGAGTCGAGGACGGATCGGTA	54
DX-3	ACTCTACCGCACCAGAATGGAG<br>TACCGATCCGTGGCTACTGTCT	22
DX-4	TACCGATCCGTGGCTACTGTCT	22
DX-2-CTG-3′FAM	AGACAGTAGCCTGCTATCTTATGGCGTGGCAAATGAGTCGAGGACGGATCGGTATTCT<br>GCTGCTGCTGT/36-FAM/	54
DX-2-T6-3′FAM	AGACAGTAGCCTGCTATCTTATGGCGTGGCAAATGAGTCGAGGACGGATCGGTATTCT<br>GCTGTTTTTTT/36-FAM/	54
DX-2-T12-3′FAM	AGACAGTAGCCTGCTATCTTATGGCGTGGCAAATGAGTCGAGGACGGATCGGTATTTT<br>TTTTTTTTTTT/36-FAM/	54
DX-4-CAG-3′IB	TACCGATCCGTGGCTACTGTCTTTCAGCAGCAGCAGT/3IABkFQ/	22
$(CAG)_4$	TCAGCAGCAGCAGT	14

Supplementary Table 2. ITC thermodynamic parameters of switchback and conventional duplex.

Parameter	Conventional duplex	Switchback DNA
Kd	1.29 ± 0.8 nM	30.73 ± 6.2 nM
n	1.01	0.94
ΔH	-83.33 ± 3.8 kcal/mol	-67.61 ± 1.8 kcal/mol
$-T\Delta S$	71.10 ± 4.2 kcal/mol	57.35 ± 1.9 kcal/mol
ΔG	-12.23 ± 0.5 kcal/mol	-10.26 ± 0.1 kcal/mol

Supplementary Table 3. Thermal melting analysis of structures containing mismatches.

	Conventional duplex		Switchback DNA	
	T_m (°C)	ΔT_m (°C)	T_m (°C)	ΔT_m (°C)
No mismatch	66.5 ± 1.3	-	41.7 ± 1.0	-
1 mismatch	58.5 ± 1.5	8.5	36.7 ± 1.5	5.0
2 mismatches (adjacent)	54.3 ± 2.0	12.1	25.2 ± 0.3	16.5
2 mismatches (separated)	44.3 ± 1.2	22.1	32.8 ± 0.8	8.8

Supplementary Table 4. Enzymes and reaction components used in biostability analysis.

Nuclease	Cat #	Reaction components	Vendor
DNase I	M0303S	DNase I reaction buffer	New England Biolabs
T5 Exo	M0663S	NEBuffer 4	New England Biolabs
Exonuclease V (Rec BCD)	M0345S	NEBuffer 4, ATP	New England Biolabs
Exonuclease VIII	M0545S	NEBuffer 4	New England Biolabs
Micrococcal nuclease	M0247S	Micrococcal nuclease reaction buffer, recombinant albumin	New England Biolabs

Supplementary Table 5. Calculated energy components that contribute towards the enthalpy change (ΔH) calculation using the MMPBSA method for the duplex and switchback DNA formed by CAG repeat sequences.

Energy Component	Conventional duplex	Switchback DNA
ΔE_{VDW}	−61.07 ± 5.7	−58.91 ± 6.1
ΔE_{EL}	2037.78 ± 38.4	2168.46 ± 66.9
ΔE_{MM}	1976.72 ± 37.6	2109.55 ± 65.4
ΔG_{PB}	−2071.19 ± 36.3	−2191.53 ± 62.5
ΔG_{NP}	-6.41 ± 0.2	−7.06 ± 0.3
ΔG_{SOL}	−2086.5967 ± 36.4	−2198.59 ± 62.6
ΔH	−100.89 ± 4.8	−89.04 ± 5.7

Supplementary Table 6. Primer sequences used for RT-qPCR.

Gene	Forward	Reverse
CXCL-8	TGTCTGGACCCCAAGGAA	CATCTTCACTGATTCTTGGATACC
CCL5	GAGTATTTCTACACCAGTGGCA	GACTCTCCATCCTAGCTCATCT
IFIT1	CCACAAGACAGAATAGCCAGAT	GCTCCAGACTATCCTTGACCT
GAPDH	CACGTTTTGGATGCACTGAGAC	GATGGAGGGCCTTTTATTCGCG
